# Case of Pregnancy and Labor Attended with Convulsions, and Complicated with Many Anomalous Symptoms, Terminating Fatally

**Published:** 1832

**Authors:** John L. Richmond

**Affiliations:** Cincinnati


**Art. V.** *Case of Pregnancy and Labor attended with Convulsions, and complicated with many anomalous symptoms, terminating fatally;* Reported by John L. Richmond, M. D. of Cincinnati.

Being called on the 7th of February, 1831, to visit a Mrs. C. I found her laboring under the most exquisite pain in the head I ever saw any person, perhaps, in my life. The pain was intermittent, and between the paroxysms, which continued only from one to two minutes, she was comparatively easy. The intermissions were, perhaps, twice as long as the paroxysms.

It was about the 7th month of the third pregnancy; she was about 24 years of age, had a good constitution, and, generally speaking, had enjoyed good health. During her previous pregnancies she had suffered but very little inconvenience, and had had easy labors. Finding her pulse hard and wiry, I extracted, from the arm about twenty ounces of blood, which was attended with a very favorable effect. Finding that the bowels were rather constipated, I ordered an active cathartic, to be assisted by enema, and directed the pediluvium and sinapisms to the feet.

On returning in the evening, I found my patient much better; gave her an anodyne and directed the sinapisms renewed. The next morning she was much revived and was comparatively easy. I directed the bowels to be kept soluble with laxatives, and advised a spare diet, hoping she would entirely recover. She continued comparatively well until the twenty-sixth, when she was taken in the same manner again, only not as severely. I extracted blood again, but with less effect than before; the bowels were now regular, and as there was no indication of the necessity of purgatives, I resorted to the use of opium, which produced a favorable effect, though it was necessary to use large doses. At this time she manifested some appearance of premature labor, but in the course of twenty-four hours became easy. She was accustomed to an active life, though not under the necessity of laboring. I advised an easy life and a light diet, with particular directions to keep the bowels in a natural state. I saw her several times in the course of a week, during which time she complained of some heaviness in the abdomen, and some pain in the loins; but she attended to the concerns of her family, and rode out several times a short distance.

On the 7th of March, her husband called on me in great haste, and informed me that she was suddenly, and violently taken in labor, and that he feared I would be too late. I hastened to her chamber, and found her, apparently, in the most severe labor pain, and really expected to find the child escaping from the last passages; but on putting my hand into the bed, was surprised to find that not the least effect had been produced, and that the os uteri remained undilated and undilatable.

I administered a free anodyne, and repeated it in an hour, which again procured ease. From this time, however, she continued in a continual state of uneasiness, and enjoyed no entire repose. She was continually harassed with extraordinary motions of the fœtus, which seemed, sometimes, to perform entire revolutions. She complained of frequent pains in the head and loins; indeed, she was seldom clear of one or the other; and it was remarkable, that when it was in the head, there was no appearance of labor-pains, and this alternate affection continued throughout. Thus she continued until the 29th of March, when she was again taken with all the appearances of violent labor. The pulse being rather hard, and finding the os uteri undilated, I detracted about 12 ounces of blood, and after waiting for several hours, and finding that the labor did not progress to any advantage, I administered an anodyne of sufficient strength to procure comparative rest during the night.

During the 30th and 31st, she continued in a very uncomfortable situation, sometimes laboring under severe pain in the head, at other times, experiencing considerable labor-pains; during which time a moderative purgative was administered, the pediluvium, and sinapisms. On the first of April she was more comfortable than she had been for several weeks. I saw her in the afternoon, and she was quite cheerful.

About nine in the evening, her husband called on me in the greatest haste, and informed me that she was in convulsions. I hurried to the house with all possible speed, and found her in a state of profound coma, from which, in a few minutes, she passed into a distressing fit of epilepsy, which continued for a space of two or three minutes, and then subsided, leaving her comatous again. The pulse being full and hard, I immediately opened a vein in the arm, and bled her freely, after which she had but one fit: and feeble irregular labor-pains ensued.

Drs. Staughton and Lawrence were immediately called in consultation. The warm bath was resorted to, with cool applications to the head, after which she became relaxed and feeble; the bowels were freely moved by cathartics and enemata.

After the convulsions there appeared no evidences of life in the child; the labor-pains increased gradually during the night, though the patient manifested but very little sense of any thing. Dr. Staughton continued with me until near morning, when it was concluded, that if the labor could be increased, there was reason to believe that it might relieve the head, and the os uteri being now somewhat dilated, it was agreed to give her a dose of Ergot, which was accordingly done. This produced the desired effect to a certain extent, as it increased the labor for a time, during which the head was obviously relieved, but the effect was not sufficient to expel the fœtus, nor even to overcome, to any extent, the rigidity of the os uteri; and, as soon as the effect ceased, the head became much affected, and the patient more insensible again.

On the morning of the 2d, as it was impossible to apply the forceps to the head, or to introduce the hand, and there had been no motion of the child for several hours, and it appearing almost certain that the child was dead, it was agreed that the head of the child should be perforated, and the crotchet applied. This was effected with but little difficulty, but when the extracting force was applied, the child being yet in utero, it was found difficult to gain much ground, the os uteri, however, became sufficiently dilated, after considerable of an effort, to admit of the introduction of the hand; and as during the time that the extractive force was applied, the head seemed considerably relieved, it was hoped that, if delivery could be effected, it would afford permanent relief. I therefore introduced my hand, turned the child, and delivered by the feet.

During the introduction of the hand, which was attended with but little difficulty, the uterus assumed considerable of a contractile power, and during the act of turning and delivering the patient manifested considerable sensibility, the uterus performed a number of very natural throes, and we became much encouraged. The placenta became entirely detached from the uterus, and was readily expelled, with very little assistance, and for a space of time every countenance was brightened with the hopes that were inspired by the more favorable appearances of our afflicted patient. But, these hopes were transient, for our patient soon sunk, again, into a state of insensibility, and our expectations appeared to be but badly grounded. We then applied a large blister to the region of the abdomen, and continued the cold applications to the head and the warm ones to the feet, which had been used for a number of hours. When the blister began to act, our hopes were again revived for a season. Some after pains took place, and the head appeared to be much relieved, but all this was to no purpose. As soon as the blister was done drawing, every thing returned again to its most gloomy form. Our patient from this time became more insensible, sank slowly, but surely, and about eleven o'clock at night expired. Various other means were employed to prevent her from sinking, but all to no effect. What cause could be rendered for the uncommon phenomena of this case? Wherein was the error, and what the deficiency of practice.

If I should meet with another case of the same kind, I should administer opium in full narcotic doses, previous to the spasms or epileptic fits; after that I know of no alteration which I could propose.

